# Comparison of OSCE performance between 6- and 7-year medical school curricula in Taiwan

**DOI:** 10.1186/s12909-021-03088-7

**Published:** 2022-01-04

**Authors:** Jr-Wei Wu, Hao-Min Cheng, Shiau-Shian Huang, Jen-Feng Liang, Chia-Chang Huang, Ling-Yu Yang, Boaz Shulruf, Ying-Ying Yang, Chen-Huan Chen, Ming-Chih Hou, Wayne Huey-Herng Sheu

**Affiliations:** 1 Department of Medical Education, Taipei Veterans General Hospital and College of Medicine, National Yang-Ming Chiao Tung University, Taipei, Taiwan; 2 College of Medicine, National Yang Ming Chiao Tung University, Taipei, Taiwan; 3 Clinical Innovation Center, Taipei Veterans General Hospital, Taipei, Taiwan; 4 Center for Evidence-based Medicine, Taipei Veterans General Hospital, Taipei, Taiwan; 5 Division of Clinical Skills Training Center, Taipei Veterans General Hospital, Taipei, Taiwan; 6 University of New South Wales, Sydney, Australia; 7 Division of Gastroenterology and Hepatology, Department of Medicine, Taipei Veterans General Hospital, Taipei, Taiwan; 8 Section of Endocrinology and Metabolism, Department of Medicine, Taipei Veterans General Hospital, Taipei, Taiwan; 9 Institute of Medical Technology, College of Life Science, National Chung-Hsing University, Taichung, Taiwan

**Keywords:** Curricular reform, Curricular length, OSCE, Clinical skills, Sub-internship, Competency-based medical education

## Abstract

**Background:**

The year 2013 marks a watershed in the history of medical education in Taiwan. Following Taiwan’s Taskforce of Medical School Curriculum Reform recommendations, the medical school curriculum was reduced from 7 to 6 years. This study aimed to analyze the impact of medical school curriculum reform on medical students’ performance in objective structured clinical examinations (OSCEs).

**Methods:**

We retrospectively analyzed the OSCE records at Taipei Veterans General Hospital (Taipei VGH), one of Taiwan’s largest tertiary medical centers, between November 2016 and July 2020. The eligibility criteria were medical students receiving a full one-year clinical sub-internship training at Taipei VGH and in their last year of medical school. All medical students received a mock OSCE-1 at the beginning of their sub-internship, a mock OSCE-2 after six months of training, and a national OSCE at the end of their sub-internship. The parameters for performance in OSCEs included “percentage of scores above the qualification standard” and “percentage of qualified stations.”

**Results:**

Between November 2016 and July 2020, 361 undergraduates underwent clinical sub-internship training at Taipei VGH. Among them, 218 were taught under the 7-year curriculum, and 143 were instructed under the 6-year curriculum. Based on baseline-adjusted ANCOVA results, medical students under the 7-year curriculum had a higher percentage of scores above the qualification standard than those under the 6-year curriculum at the mock OSCE-1 (7-year curriculum vs. 6-year curriculum: 33.8% [95% CI 32.0–35.7] vs. 28.2% [95% CI 25.9–30.4], *p* < 0.001), and mock OSCE-2 (7-year curriculum vs. 6-year curriculum: 89.4% [95% CI 87.4–91.4] vs. 84.0% [95% CI 81.5–86.4], *p* = 0.001). Moreover, medical students in the 7-year curriculum had a higher percentage of qualified stations in mock OSCE-1 (7-year curriculum vs. 6-year curriculum: 89.4% [95% CI 87.4–91.4] vs. 84.0% [95% CI 81.5–86.4], *p* = 0.001) and mock OSCE-2 (7-year curriculum vs. 6-year curriculum: 91.9% [95% CI 90.1–93.8] vs. 86.1% [95% CI 83.8–88.3], p = 0.001). After clinical sub-internship training, there were no differences in the percentage of scores above the qualification standard (7-year curriculum vs. 6-year curriculum: 33.5% [95% CI 32.2–34.9] vs. 34.6 [95% CI 32.9–36.3], *p* = 0.328) and percentage of qualified stations (7-year curriculum vs. 6-year curriculum: 89.4% [95% CI 88.1–90.7] vs. 90.2% [95% CI 88.6–91.8], *p* = 0.492).

**Conclusions:**

At the beginning of the sub-internship, medical students under the 7-year curriculum had better OSCE performance than those under the 6-year curriculum. After the clinical sub-internship training in Taipei VGH, there was no difference in the national OSCE score between the 6- and 7-year curricula. Our study suggests that clinical sub-internship is crucial for the development of clinical skills and performance in the national OSCE.

## Introduction

**Fig. 1:**
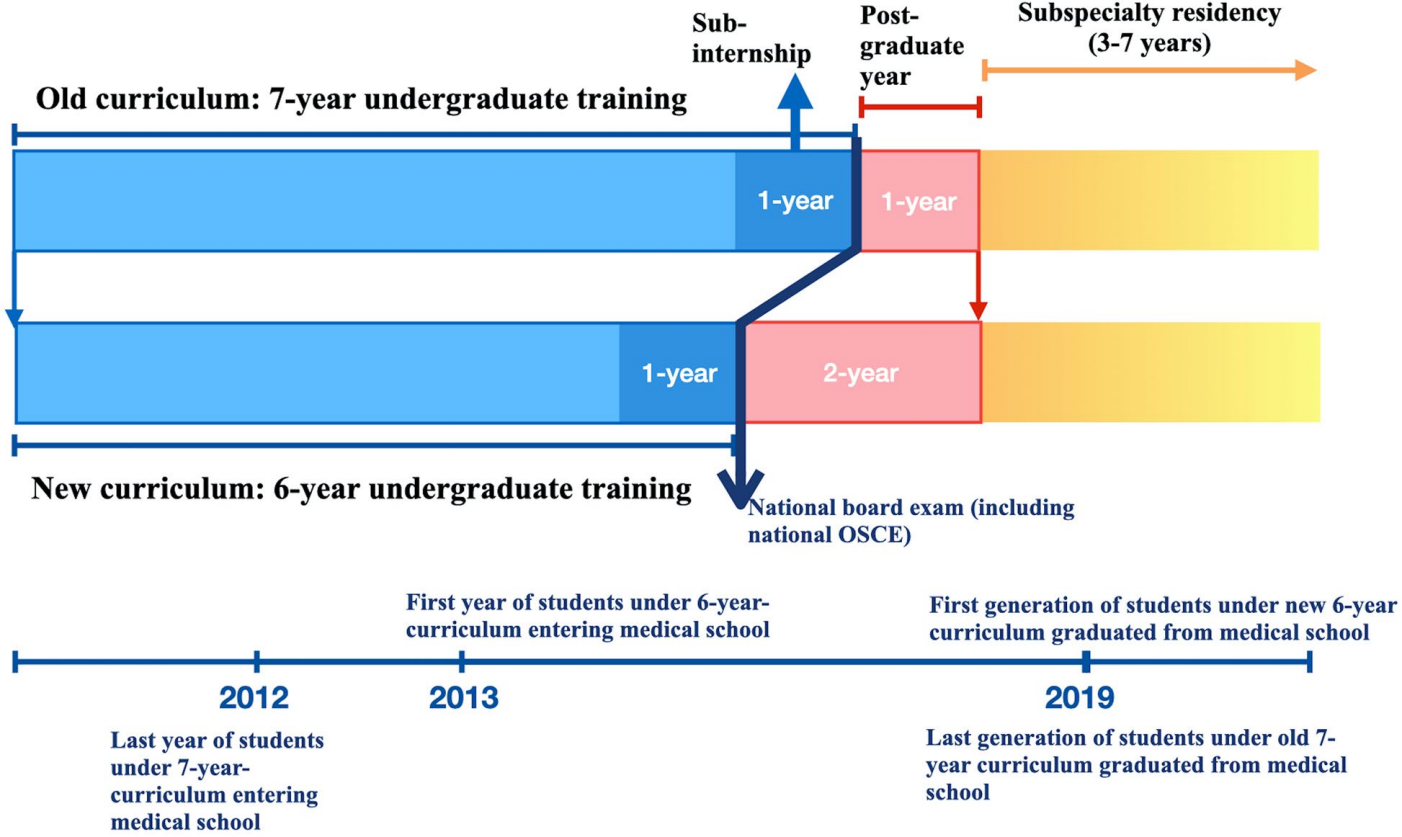
Curricular reform in Taiwan

Curricular reform in medical schools has become a popular topic in recent decades [1–3]. In Taiwan, the length of the medical school curriculum has been shortened from seven to six years, following the recommendations of Taiwan’s Taskforce of Medical School Curriculum Reform [4]. The primary goal of Taiwan’s curricular reform is to strengthen clinical training and ensure that medical students can enter clinical training earlier. The curricular reform did not allow students to make choice between 6 and 7-year-curricula at the same time (Fig. 1). Both 7- and 6-year curricula have clinical sub-internship training in the last year of medical school. The old 7-year-curriculum included seven years of undergraduate training (including sub-internship training in the last year), and 1-year post-graduate training. After the curricular reform, the 6-year-curriculum include six years of undergraduate training (including sub-internship training in the last year), and an extended 2-year post-graduate training (Fig. 1) [5]. The central philosophy behind the curriculum reforms is John Dewey’s “learning-by-doing theory.” [5] The “learning-by-doing theory” includes cultivating problem-solving and task-achievement abilities by using project-based learning and encouraging students to engage in lifelong learning. Thus, the core driving force of Taiwan’s curricular reform is to improve educational outcomes.

Shortening the medical curricula is an international phenomenon. For example, in the United States, there have been three waves of shortening the curricular length of medical schools. In the United States, a 3-year curriculum was introduced in the 1940s and 1970s [6]. In the 1940s, the 3-year curriculum was introduced to compensate for the physician shortage [6]. In contrast, the 3-year curriculum in the 1970s was introduced due to financial considerations, which included reducing student debt and providing government financial incentives [7]. However, the prevalence of the 3-year curriculum declined due to the discontinuation of government funding [7]. Nowadays, some medical schools in the United States such as the New York University School of Medicine offer a 3-year MD program [8]. In the United States, the reasons for curriculum changes in the 1940s and 1970s are not the outcome of medical education but the result of societal demands or shortage of phyisicans [9, 10]. Recently, a third wave of curricular reform called “accelerated programs” has been proposed by 35% of medical schools in the United States [11]. Most of these accelerated programs are usually combined with a partner graduate medical education program [12]. However, at present, the educational outcome of the accelerated programs remains an unanswered question [9]. Hence, the experience of Taiwan’s curricular reform can provide medical educators a valuable opportunity to compare the clinical competency between different curricula of different lengths. The purpose of this study was to analyze the clinical performance between 6- and 7-year curricula using standardized serial Objective Structured Clinical Exams (OSCEs).

## Method

We retrospectively analyzed the data of mock and national OSCEs of undergraduates at Taipei Veterans General Hospital (VGH), a tertiary medical center in Taiwan, between November 2016 and July 2020. All undergraduates received two mock OSCEs and one national OSCE during their sub-internships. The mock OSCE-1 and mock OSCE-2 are given two months and six months after the beginning of the sub-internship, respectively. Finally, students take the national OSCE in the last month of training.

### The procedure of OSCEs

The procedure of OSCEs has been validated and widely applied in Taiwan’s teaching hospital for more than ten years [13]. All examinees are given the same problem and asked to execute the same task [14]. The examiners evaluate the examinees’ performance based on a standardized checklist. The standardized patient should be trained by healthcare professionals to act as a patient according to the standardized role play script [15]. In Taipei VGH, mock OSCEs have six stations and 12 stations for national OSCEs (due to logistical concerns), with each station taking eight minutes. There is no difference in the requirements of raters, standard patients, and examination space between the mock and national OSCEs. Both mock and national OSCEs include various clinical skills related to internal medicine, surgery, pediatrics, obstetrics, gynecology, and emergency medicine. Apart from different medical specialties, the whole OSCE should cover the competencies of history taking, communication skills, procedural skills, physical examination, differential diagnosis, and clinical reasoning. In each station, each examinee received scores from the checklist and global rating scores. The score of each item from the checklist includes 0 (not at all), 2 (partially achieved), and 3 (completely achieved). The total items from the checklist of each station could be different; hence, scores from the checklist transformed into the percentage of the total scores of each station. The global rating score in each station can be divided into five levels, which include 1 (poor), 2 (fair), 3 (average), 4 (good), 5 (excellent). In both the mock and national OSCEs, the examinee-centered borderline group method with regression is applied to establish a passing standard [16–18]. The passing standard of each station is the mean scores from the checklist in those rated level 2 in the global rating.

### Outcome measurement of OSCEs

In this study, we used two outcome measurements for medical students’ OSCE performance. The percentage of scores above the qualification standard is defined as the difference between the students’ actual score and the approval standard score, divided by the approval standard score. In Taiwan, the determination of the approval standard score is based on the examinee-centered borderline group method with regression, following the standard protocol of Taiwan’s national OSCE board [16, 18–23]. This study also introduced the percentage of qualified stations in the OSCE as another outcome measurement.

### Statistics

Relationships were analyzed between outcome measurements and different curricula, as well as demographics. Means were reported with standard deviations, and medians were reported with interquartile ranges. Comparisons of performance in serial OSCE were analyzed using repeated-measures ANOVA. To compare the OSCE performance between the two curricula, a two-way analysis of covariance (ANCOVA) for repeated measures was performed with the baseline scores used as a covariate to eliminate the possible demographic influence of OSCE performance [24]. Relationships were analyzed between different curricula using chi-square and t-tests as appropriate. For all analyses, results were considered significant at *p* < 0.05. All statistical analyses were conducted using IBM SPSS (version 22.0).

## Results

### Demographics and baseline characteristics

Between November 2016 and July 2020, 361 undergraduates (132 female and 229 male) underwent clinical internship training at Taipei VGH. Among them, 218 were under the 7-year curriculum, and 143 were under the 6-year curriculum. Comparing the two curriculum lengths, students under the 6-year curriculum had a higher percentage of clerkship training at our institution (7-year curriculum vs. 6-year curriculum: 67.0% vs. 89.5%, *p* < 0.001) (Table 1). Based on repeated ANOVA, there is a significant improvement from the beginning of training (mock OSCE-1), after six months of training (mock OSCE-1), and at the end of the training (national OSCE) in the percentage of scores above the qualification standard (%, mean ± SD) (OSCE-1 vs. OSCE-2 vs. national OSCE: 31.6 ± 14.6% vs. 29.8 ± 12.4% vs. 34.0 ± 10.2%, *p* < 0.001), and the percentage of qualified stations (OSCE-1 vs. OSCE-2 vs. national OSCE: 87.3 ± 15.3% vs. 89.6 ± 14.0% vs. 89.7 ± 9.8%, *p* = 0.005) (Table 2).

**Table 1:** Demographics and descriptive statistics between 6 and 7-year curriculum

Variables	7-year curriculum (*N* = 218)	6-year curriculum (*N* = 143)	*p*-value*
Sex (n, %)			
Men	141 (64.7%)	88 (61.5%)	0.545
Women	77 (35.3%)	55 (38.5%)	
Students received clerkship at our institution (n,%)	146 (67.0%)	128 (89.5%)	< 0.001*
Trainees’ medical school (n, %)			
National Yang-Ming University	146 (67.0%)	128 (89.5%)	< 0.001*
Taipei Medical University	20 (9.2%)	0 (0.0%)	< 0.001*
Kaohsiung Medical University	1 (0.5%)	0 (0.0%)	0.417
Chung Shan Medical University	31 (14.2%)	0 (0.0%)	< 0.001*
Fu Jen Catholic University	3 (1.4%)	0 (0.0%)	0.159
China Medical University	8 (3.7%)	0 (0.0%)	0.021*
National Defense Medical Center	9 (4.1%)	15 (10.5%)	0.018*
Percentage of score above the qualification standard (mean ± SD%)			
Mock OSCE-1	32.7 ± 13.7%	29.7 ± 15.6%,	0.051
Mock OSCE-2	33.9 ± 12.1%	23.6 ± 10.3%	< 0.001*
National OSCE	33.2 ± 10.8	35.1 ± 9.2%,	0.081
Percentage of qualified stations (mean ± SD%)			
Mock OSCE-1	88.7 ± 14.6%	85.1 ± 16.2%,	0.028*
Mock OSCE-2	91.7 ± 12.3%	86.4 ± 15.8%	0.001*
National OSCE	89.2 ± 10.6%	90.6 ± 8.5%	0.175

*Results were considered significant by p < 0.05

**Table 2:** The performance in serial OSCEs

Outcome measurements (mean ± SD)		Mock OSCE-1 (Beginning of sub-internship)	Mock OSCE-2 (6th month of sub-internship)	National OSCE (11th month of sub-internship)	*p*-value*
Percentage of score above the qualification standard (mean ± SD%)	7-year curriculum	32.7 ± 13.7%	33.9 ± 12.1%	33.2 ± 10.8%,	*p* = 0.505
	6-year curriculum	29.7 ± 15.5%	23.6 ± 10.3%	35.1 ± 9.2%,	p < 0.001
	Total (6 and 7-year)	31.6 ± 14.6%	29.8 ± 12.4%	34.0 ± 10.2%,	*p* < 0.001
Percentage of qualified stations (mean ± SD%)	7-year curriculum	88.7 ± 14.5%	91.7 ± 12.3%	89.2 ± 10.6%,	0.009
	6-year curriculum	85.1 ± 16.2%	86.4 ± 15.8%	90.6 ± 8.5%,	< 0.001
	Total (6 and 7-year)	87.3 ± 15.3%	89.6 ± 14.0%	89.7 ± 9.8%,	0.005

*Results were considered significant by p < 0.05

### Factors associated with OSCE performance

At Taipei VGH, most (77%) undergraduate students received clerkship training at our institution. Among baseline demographics, history of received clerkship at our institution was associated with better performance in OSCEs in two outcome measurements (Table 3).

**Table 3:** Association between history clerkship training at our institution and OSCEs Performance

Variables	Received clerkship training at our institution (*N* = 274)	Not received clerkship training at our institution (*N* = 87)	*p*-value*
Percentage of score above the qualification standard (mean ± SD%)			
Mock OSCE-1	33.9 ± 13.9%	24.4 ± 14.4%,	< 0.001*
Mock OSCE-2	29.8 ± 11.9%	29.9 ± 14.1%	0.984
National OSCE	34.8 ± 10.0%	31.3 ± 10.5%	0.005*
Percentage of qualified stations (mean ± SD%)			
Mock OSCE-1	88.7 ± 13.6%	82.6 ± 19.0%	0.006*
Mock OSCE-2	89.6 ± 13.9%	89.7 ± 14.4%	0.974
National OSCE	90.4 ± 9.1%	87.7 ± 11.7%	0.058

*Results were considered significant by p < 0.05

### Comparing OSCE performance between two curriculum lengths

**Fig. 2:**
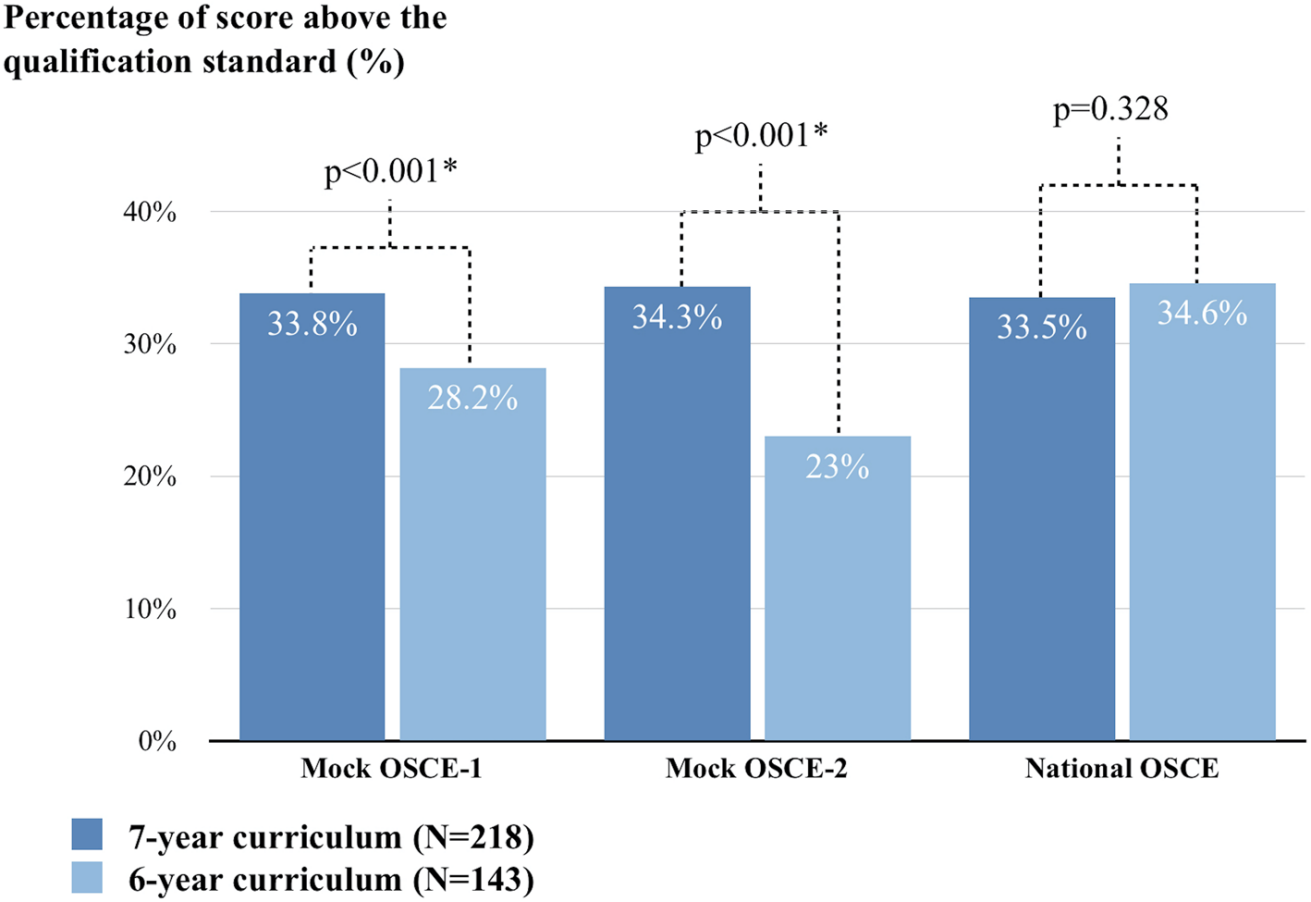
The difference in percentage of score above the qualification standard (%) between 7-year and 6-year curriculum after adjusted confounding factors

**Fig. 3:**
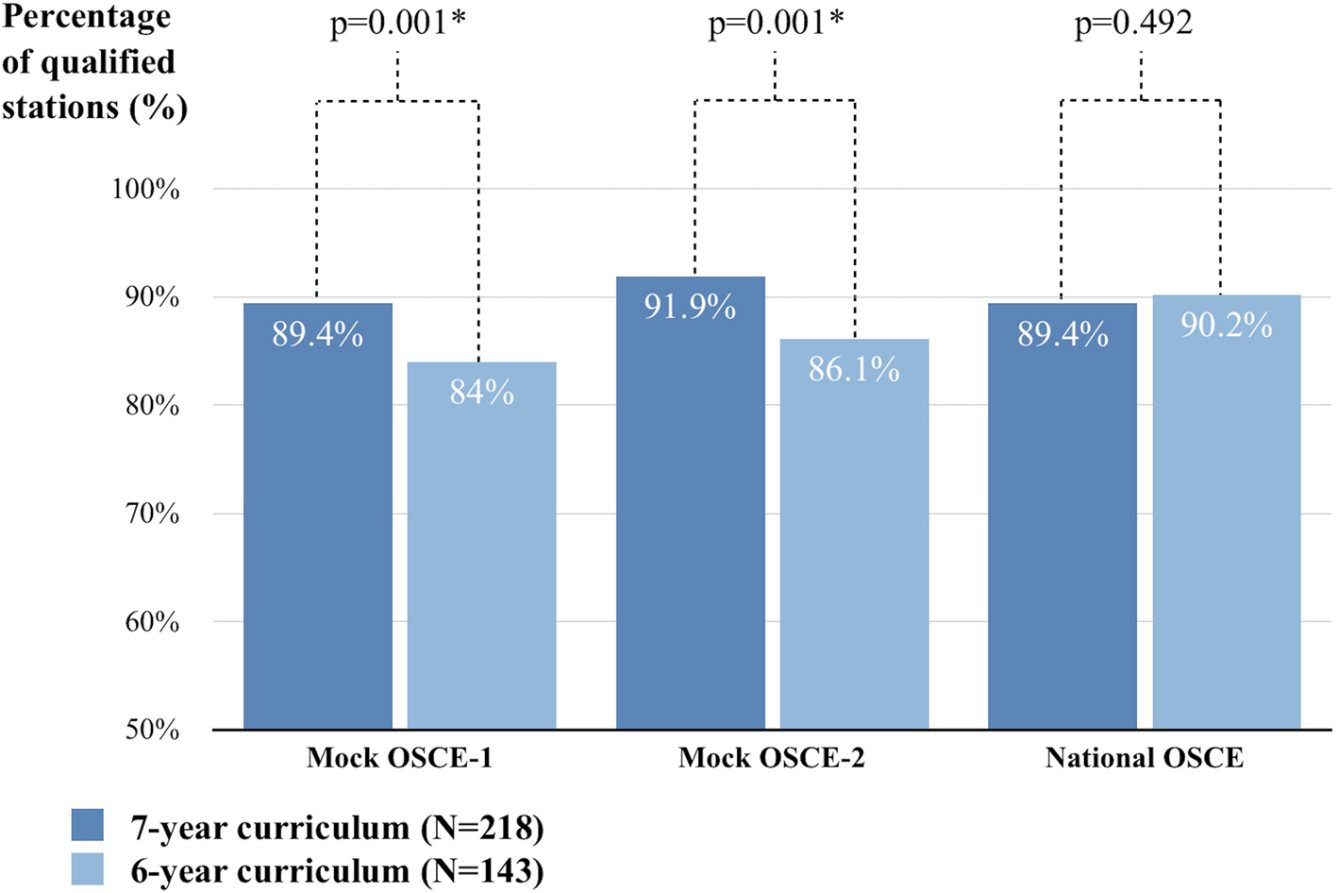
The difference in percentage of qualified stations (%) between 7-year and 6-year curriculum after adjusted confounding factors

To exclude the potential confounding effects of history of received clerkship at our institution, the comparisons of OSCE outcomes between 6- and 7-year curricula were based on baseline-adjusted ANCOVA results. In the mock OSCE-1, medical students under the 7-year curriculum had a higher percentage of scores above the qualification standard (mean [95% confidence interval]) than those under the 6-year curriculum (7-year curriculum vs. 6-year curriculum: 33.8% [95% CI 32.0–35.7] vs. 28.2% [95% CI 25.9–30.4], *p* < 0.001), and a higher percentage of qualified stations (7-year curriculum vs. 6-year curriculum: 89.4% [95% CI 87.4–91.4] vs. 84.0% [95% CI 81.5–86.4], *p* = 0.001). In the mock OSCE-2 (after six months of training), medical students under the 7-year curriculum had a higher percentage of scores above the qualification standard than those under the 6-year curriculum (7-year curriculum vs. 6-year curriculum: 34.3% [95% CI 32.8–35.8] vs. 23.0 [95% CI 32.8–35.8], *p* < 0.001), and a higher percentage of qualified stations (7-year curriculum vs. 6-year curriculum: 91.9% [95% CI 90.1–93.8] vs. 86.1% [95% CI 83.8–88.3], *p* = 0.001). In the national OSCE, there were no differences in the percentage of scores above the qualification standard (7-year curriculum vs. 6-year curriculum: 33.5% [95% CI 32.2–34.9] vs. 34.6 [95% CI 32.9–36.3], *p* = 0.328) and the percentage of qualified stations (7-year curriculum vs. 6-year curriculum: 89.4% [95% CI 88.1–90.7] vs. 90.2% [95% CI 88.6–91.8], *p* = 0.492) (Figs. 2 and 3).

## Discussion

In this study, we found medical students under the 7-year curriculum performed better in OSCE than their 6-year curriculum counterparts at the beginning of the internship. After the clinical internship training at Taipei VGH, there was no difference in national OSCE scores between the 6- and 7-year curricula graduates.

There is a paucity of controlled studies comparing the outcomes between different medical school curriculum lengths [9]. Therefore, the long-term experience of medical curriculum reform in Canada provides us with a useful window to observe its impact on physicians’ performance and competency. In Canada, there has continued 3-year programs at both the University of Calgary and McMaster University since the 1970s. By using questionnaire scores from colleagues and patients, a 30-year long-term observation in Alberta showed that physicians of 3-year programs (from the University of Calgary) do not seem to be less competent than those who graduate from 4-year programs [25]. The present study findings are similar to those of Canada; that is, a shorter medical school curriculum seems to not interfere with the development of students’ clinical competence. Recently, some medical schools in the United States with 3-year MD programs introduced UME-GME continuum programs at their own institutions to strengthen the clinical competency of residents with a shorter curriculum [1]. In the present study, we found that students who received a clerkship at the same institution had a trend of better OSCE performance, suggesting that continuum programs can help accelerate clinical competencies. One reason for the better OSCEs performance in those who received ‘continuum programs’ (clinical clerkship and sub-internship at our institution) includes the ‘home advantage.’ This subgroup of trainees had built up their competencies of ‘system-based practice’ during their clerkship and could spend more time acquiring other clinical competencies in their sub-internship. Also, features of our curricular design for clinical clerkship could be another explanation. Our clinical clerkship compromised 80 important competencies with the well-designed training program. Trainees could receive clinical training in several departments, including 3-month internal medicine, 3-month surgery, 1.5-month OBS-GYN, 1.5-month pediatrics, 1-month neurology and psychiatry, 0.5-month orthopedics, and 0.5-month geriatrics. The diverse learning scenario ensures trainees have more experience in dealing with the different clinical situations.

The strength of the present study is that we used standardized OSCEs as outcome measurements. This OSCE protocol has been integrated into the national medical examination in Taiwan, which is a more objective measurement for the educational outcome [26]. Another strength is that the present study further analyzed the performance in serial OSCEs, thus providing a more comprehensive and longitudinal viewpoint for internship training. According to our research findings, medical students under a shorter curriculum have lower OSCE scores at the beginning of their internship, but there is no difference between 6- and 7-year curricula outcomes after the internship training. There are four possible explanations for these findings. First, the clinical teachers had information about students under different curriculum lengths. Therefore, clinical teachers may devote more effort to students in a shorter curriculum. Also, our institution organized a task force to strengthen the development of clinical competencies for trainees under the shortened curriculum. Strategies such as specialized mini-lectures, ongoing supportive supervision, and clinical mentorship have been implanted, which may contribute to the ‘catch-up’ of sub-interns under a 6-year-curriculum. Second, we introduced the competency-based medical education (CBME) framework simultaneously with the curricular reform. Under the new curriculum, undergraduates had been informed about the required competencies after completing their training a priori starting their sub-internship at our institution. Also, we redesigned our electronic assessment system and introduced a CBME-based evaluation framework for trainees under the new 6-year-curriculum. Hence, the length of the curriculum is no longer the key factor for educational outcomes [27]. Third, the lack of growth in the 7-year curricula graduates may be due to the ceiling effect; that is, most students achieved the goal of clinical sub-internship training. The measurement of advanced competencies for real-world practice is beyond the scope of OSCEs, which may require direct observation in the clinical setting. Fourth, our study found inferior performance in the initial mock-OSCE from those under the 6-year curriculum. The shortening lectures for clinical medicine before entering sub-internship may be the reason for inferior performance in the 1st mock-OSCE (at the beginning of sub-internship) from those under the 6-year curriculum. Moreover, our findings show that the clinical setting is the best strategy for efficient learning, which echoes the philosophy behind the curriculum reforms, that is, John Dewey’s “learning-by-doing theory.”

One critical issue that should be addressed is the decline of the 2nd OSCE shown in Table 2. The reasons underlying this decline might be due to learning fatigue or distraction. The distraction could be attributed to sub-interns focusing on applying for post-graduate training programs simultaneously as 2nd OSCE. The present study provides us a critical chance to improve the curricula design to prevent learning fatigue and distraction. Also, our study had several limitations. First, the present study was not controlled, and several possible confounding factors may have interfered with our results. For example, clinical teachers and institutions may change their teaching strategies after implementing a 6-year curriculum. Since it is nearly impossible to conduct a randomized controlled study to compare the outcomes of different curriculum lengths, the curriculum reform in Taiwan can provide us with an opportunity to analyze the association between curriculum length and clinical competency. Second, the study did not control for underlying school performance before entering the sub-internship. In Taiwan, medical students do not provide their grade reports to their sub-internship hospitals, and medical students from different medical schools may have different standards for academic grading. However, as every sub-intern in our institution took the mock OSCE-1 at the beginning of their sub-internship their initial scores could provide a standardized measurement for their educational outcome before entering the clinical sub-internship. Third, there are differences between the percentage of certain medical schools between trainees under 6- and 7-year-curriculum. The differences between the percentage of several medical schools were due to the increased capacity of certain university hospitals. Thus, their students can receive their sub-internship at their own university hospital. This is the reason for the decreased proportion of some medical schools between 6- and 7-year-curriculum. Fourth, the pass rate of national OSCEs is generally higher in our institution than the national average pass rate (Table 4) [28]. Therefore, caution should be taken to generalize our results into trainee at different institutions. Further studies using the national cohort is needed to portray the landscape of impacts after curricula reform in Taiwan.

**Table 4:** The OSCE pass rate of our institution and national average of Taiwan

Year	Our institution (%)	National average (%)
2020	100%	98.78%
2019	100%	98.23%
2018	100%	98.55%
2017	97.82%	99.01%

The statistics are not available because the authors cannot obtain the original data from Ministry of Examination of Taiwan

## Conclusions

At the beginning of the sub-internship, medical students under the 7-year curriculum outperformed those under the 6-year curriculum on the OSCE. After the sub-internship training, there was no difference in the national OSCE score between the 6- and 7-year curricula. Our study showed that changes in curriculum length in medical schools did not interfere with the OSCE performance at the end of the sub-internship, and clinical training is a crucial factor for developing clinical competencies. Our experience can inspire future curriculum reforms in medical schools.

## Data Availability

Data would be available by contacting the corresponding author.

## Abbreviations

OSCE: Objective Structured Clinical Examination
ANOVA: Analysis of variance
ANCOVA: Analysis of covariance
CBME: Competency-Based Medical Education

## References

[1] Abramson SB, Jacob D, Rosenfeld M (2013). A 3-year M.D. — accelerating careers, diminishing debt. N Engl J Med.

[2] Goldfarb S, Morrison G (2013). The 3-year medical school — change or shortchange?. N Engl J Med.

[3] Emanuel EJ, Fuchs VR (2012). Shortening medical training by 30%. Jama..

[4] Chiu CH, Arrigo LG, Tsai D (2009). Historical context for the growth of medical professionalism and curriculum reform in Taiwan. Kaohsiung J Med Sci.

[5] Cheng WC, Chen TY, Lee MS (2019). Fill the gap between traditional and new era: The medical educational reform in Taiwan. Ci Ji Yi Xue Za Zhi = Tzu-Chi Medical J.

[6] Berman BU (1979). Three-year programs in medical and dental schools: an appraisal. Public Health Rep (Washington, DC : 1974).

[7] Schwartz CC, Ajjarapu AS, Stamy CD, Schwinn DA (2018). Comprehensive history of 3-year and accelerated US medical school programs: a century in review. Med Educ Online..

[8] Cangiarella J, Cohen E, Rivera R, Gillespie C, Abramson S. Evolution of an accelerated 3-year pathway to the MD degree: the experience of new York University Grossman School of Medicine. Acad Med. 2020;95(4). 10.1097/ACM.0000000000003013 31577593

[9] Raymond JR, Kerschner JE, Hueston WJ, Maurana CA (2015). The merits and challenges of three-year medical school curricula: time for an evidence-based discussion. Acad Med.

[10] Lyss-Lerman P, Teherani A, Aagaard E, Loeser H, Cooke M, Harper GM (2009). What training is needed in the fourth year of medical school? Views of residency program directors. Acad Med.

[11] Leong SL, Cangiarella J, Fancher T (2017). Roadmap for creating an accelerated three-year medical education program. Med Educ Online.

[12] Aschenbrener CA, Ast C, Kirch DG (2015). Graduate medical education: its role in achieving a true medical education continuum. Acad Med.

[13] Huang CC, Chan CY, Wu CL (2010). Assessment of clinical competence of medical students using the objective structured clinical examination: first 2 years' experience in Taipei veterans general hospital. J Chinese Med Assoc.

[14] Chong L, Taylor S, Haywood M, Adelstein BA, Shulruf B (2017). The sights and insights of examiners in objective structured clinical examinations. J Educ Eval Health Prof.

[15] Chang CC, Lirng JF, Wang PN (2019). A pilot study of integrating standardized patients in problem-based learning tutorial in Taiwan. J Chinese Med Assoc.

[16] Wood TJ, Humphrey-Murto SM, Norman GR (2006). Standard setting in a small scale OSCE: a comparison of the modified borderline-group method and the borderline regression method. Adv Health Sci Educ Theory Pract.

[17] Hejri SM, Jalili M, Muijtjens AMM, Van Der Vleuten CPM (2013). Assessing the reliability of the borderline regression method as a standard setting procedure for objective structured clinical examination. J Res Med Sci.

[18] Homer M, Pell G (2009). The impact of the inclusion of simulated patient ratings on the reliability of OSCE assessments under the borderline regression method. Med Teach.

[19] Yousuf N, Violato C, Zuberi RW (2015). Standard setting methods for pass/fail decisions on high-stakes objective structured clinical examinations: a validity study. Teach Learn Med.

[20] Norcini JJ (2003). Setting standards on educational tests. Med Educ.

[21] Dwivedi NR, Vijayashankar NP, Hansda M, et al. Comparing Standard Setting Methods for Objective Structured Clinical Examinations in a Caribbean Medical School. 2020;7:2382120520981992. 10.1177/2382120520981992 PMC7780167 33447662

[22] Kramer A, Muijtjens A, Jansen K, Düsman H, Tan L, Van Der Vleuten C (2003). Comparison of a rational and an empirical standard setting procedure for an OSCE. Med Educ.

[23] Shulruf B, Turner R, Poole P, Wilkinson T (2013). The objective borderline method (OBM): a probability-based model for setting up an objective pass/fail cut-off score in medical programme assessments. Adv Health Sci Educ Theory Pract..

[24] Van Breukelen GJ (2006). ANCOVA versus change from baseline: more power in randomized studies, more bias in nonrandomized studies [corrected]. J Clin Epidemiol.

[25] Lockyer JM, Violato C, Wright BJ, Fidler HM (2009). An analysis of long-term outcomes of the impact of curriculum: a comparison of the three- and four-year medical school curricula. Acad Med.

[26] Liu KM, Tsai TC, Tsai SL (2013). Clinical skills examination as part of the Taiwan National Medical Licensing Examination. Med Teach..

[27] Leung WC (2002). Competency based medical training: review. BMJ..

[28] 2019 Annual reports of national OSCE. Ministry of Examination of Taiwan. https://wwwc.moex.gov.tw/main/content/wHandMenuFile.ashx?file_id=2729. Accessed November 6 2021.

